# A randomised, single-blind, sham-controlled clinical trial on the effects of intermittent Theta-Burst Stimulation (iTBS) on depressive and cognitive symptoms in patients with Treatment-Resistant Depression

**DOI:** 10.1192/j.eurpsy.2026.10620

**Published:** 2026-07-31

**Authors:** L. Aita, M. G. Rossetti, D. Ricciardolo, M. Bellani

**Affiliations:** 1 UOC Psichiatria B, Azienda Ospedaliera Universitaria Integrata (AOUI) Verona; 2 Department of Neurosciences, Biomedicine and Movement Sciences, Section of psychiatry, University of Verona, Verona, Italy

## Abstract

**Introduction:**

Major Depressive Disorder (MDD) is a severe psychiatric disorder characterised by persistent low mood, anhedonia, cognitive impairment, and social cognition (S-Cog) deficits, which reduce quality of life and hinder recovery. Notably, up to 50% of patients develop Treatment-Resistant Depression (TRD), with limited therapeutic options. Transcranial magnetic stimulation has emerged as a promising treatment for TRD, with intermittent theta-burst stimulation (iTBS) showing advantages in promoting neuroplasticity while requiring shorter sessions. This study will apply this innovative medical technology to evaluate the efficacy of an iTBS protocol in improving depressive symptoms and S-Cog deficits in patients with TRD.

**Objectives:**

Forty patients with TRD will be recruited and randomised to receive either active iTBS to the lDLPFC or sham stimulation (20 sessions in 4 weeks). The iTBS protocol will be in line with the FDA guidelines. Patients will undergo extensive socio-demographic, clinical, neurocognitive/social cognitive, and functioning evaluation at baseline (T0), post-treatment (T1), and 4 weeks post-treatment (T2). Standardised questionnaires and batteries will be used for this purpose, including HDRS-21 and BDI-II (depression); SCL-90 (psychopathology); WHODAS 2.0 (functioning); SF-36 (quality of life); BAC-A (neurocognition); Reading the Mind in the Eyes test; Ekman Test, Faux Pas Test (S-Cog). Forty healthy controls (HC) will also be recruited and undergo only the clinical and cognitive/social-cognitive evaluation at T0. Data collected from HC will serve as a benchmark for interpreting the effects of active-iTBS and sham-iTBS on patients.

**Methods:**

Data will be analysed using mixed-effect models for repeated measures (T0, T1, T2); Intra-group effect sizes (Cohen’s d) will be calculated using performance change scores (mean score T1 – mean score T0) and standard deviations to quantify treatment impact within each group.

**Results:**

The primary expected outcome is an improvement of ≥50% in depressive symptoms and 30% in S-Cog, calculated as the percentage change between the mean scores at T1 and T0 on measures of depression and S-Cog. *Secondary* are - i) maintenance of the improvements at T2; ii) changes in pre-to-post measures of functioning, quality of life, and other cognitive measures;

**Conclusions:**

The project will start in November 2025 and very preliminary findings will be presented at the upcoming conference.

**Disclosure of Interest:**

None Declared

